# SLAM/SAP Decreased Follicular Regulatory T Cells in Patients with Graves' Disease

**DOI:** 10.1155/2021/5548463

**Published:** 2021-04-19

**Authors:** Lina Geng, Jun Yang, Xinyi Tang, Huiyong Peng, Jie Tian, Zhigang Hu, Yingzhao Liu, Huaxi Xu, Shengjun Wang

**Affiliations:** ^1^Department of Laboratory Medicine, Affiliated People's Hospital, Jiangsu University, Zhenjiang, China; ^2^Institute of Laboratory Medicine, Jiangsu Key Laboratory for Laboratory Medicine, Jiangsu University School of Medicine, Zhenjiang, China; ^3^Department of Laboratory Medicine, Wuxi Children's Hospital, Wuxi People's Hospital Affiliated to Nanjing Medical University, Wuxi 214023, China; ^4^Department of Endocrinology, Affiliated People's Hospital, Jiangsu University, Zhenjiang, China

## Abstract

Signaling lymphocytic activation molecule (SLAM) and SLAM-associated protein (SAP) play important role in inflammatory and autoimmune diseases. Our study is aimed at detecting the expression of SLAM and SAP in patients with Graves' disease (GD) and analyzing the effect of SLAM/SAP on circulating blood CD4^+^CXCR5^+^Foxp3^+^ follicular regulatory T (Tfr) cells. The level of SAP in CD4^+^CXCR5^+^ T cells and the level of SLAM on CD19^+^ B cells were significantly increased in the patients with GD, but no significant difference in the level of SLAM on CD4^+^CXCR5^+^ T cells was observed between the patients with GD and the healthy controls. A decrease in the percentage of Foxp3^+^ cells in CD4^+^CXCR5^+^ T cells was observed following anti-SLAM treatment, but the percentages of IFN-*γ*^+^ cells, IL-4^+^ cells, and IL-17^+^ cells showed no obvious differences. The proportion of circulating Tfr cells was decreased in the patients with GD, and the proportion of circulating Tfr cells had a negative correlation with the level of SAP in CD4^+^CXCR5^+^ T cells and the levels of autoantibodies in the serum of the patients with GD. Our results suggested that the SLAM/SAP signaling pathway is involved in the decrease of circulating Tfr cells in Graves' disease.

## 1. Introduction

Graves' disease (GD) is an organ-specific autoimmune disease with a complex pathogenesis that involves aberrant B and T lymphocyte responses. Patients with GD produce excess autoantibodies, such as anti-thyroid-stimulating hormone (TSH) receptor antibody (TR-Ab), anti-thyroperoxidase antibody (TPO-Ab), and anti-thyroglobulin antibody (TG-Ab) [1]. TR-Ab, whose positive rate in patients with untreated GD is as high as 85-100%, interacts with the TSH receptor to stimulate thyroid follicular cell hyperplasia and promote thyroid hormone synthesis and secretion [1].

The CD4^+^ T cell-mediated immune response has long been regarded as an important aspect of the pathogenesis of Graves' disease. Many studies have shown that Th1, Th2, and Th17 cells, which are subtypes of CD4^+^ T cells, are effector helper T cells that participate in the pathogenesis of Graves' disease [2–4]. However, we recently found that follicular helper T (Tfh) cells are involved in the pathogenesis of this disease [5, 6]. Tfh cells are a new subset of CD4^+^ T cells characterized by the transcription factor Bcl-6 (B cell lymphoma 6) as well as the expression of C-X-C chemokine receptor type 5 (CXCR5), inducible costimulatory molecule (ICOS), programmed cell death protein 1 (PD-1), and interleukin-21 (IL-21). In addition to generating memory B cells and plasma cells, Tfh cells are required for the formation and maintenance of the germinal center (GC). Tfh cells provide survival signals to GC B cells via multiple pathways, including CD40L, IL-4, IL-21, PD-1, signaling lymphocytic activation molecule (SLAM), and SLAM-associated protein (SAP) [7]. Our previous studies showed that the percentage of circulating Tfh cells in patients with GD was increased, which is related to the disease process [5], but the molecules and mechanisms involved in this process are unclear.

In recent years, researchers have found that peripheral blood CD4^+^CXCR5^+^ T cells can be divided into several different subgroups: Tfh1 cells mainly secrete interferon-*γ* (IFN-*γ*), Tfh2 cells mainly secrete IL-4, and Tfh17 cells mainly secrete IL-17 and IL-22 [8]. In addition, Lim and colleagues have found follicular regulatory T (Tfr) cells in the germinal center of the human tonsil, and these cells had immunosuppressive functions [9]. Although Tfr cells express some of the same markers as Tfh cells, such as Bcl-6, CXCR5, PD-1, ICOS, and SAP, Tfr cells usually develop from Treg cells but not Tfh cells [10–12]. Tfr cells can inhibit Tfh cells or induce B cell death to regulate the GC reaction [13, 14]. In contrast, Tfh cells can limit the proliferation of Tfr cells by secreting IL-21 [15, 16]. In recent years, a number of studies have reported on the immune disorders of Tfr/Tfh cells in the peripheral blood of patients with autoimmune diseases such as rheumatoid arthritis (RA), multiple sclerosis (MS), myasthenia gravis, and systemic lupus erythematosus (SLE) [17, 18].

SLAM is a type I transmembrane glycoprotein; it forms homophilic interactions and acts as a self-ligand [19–22]. SLAM is mainly expressed in thymus cells, B cells, activated T cells, dendritic cells, macrophages, and activated mononuclear cells [23–25]. Stimulating TCR activation can induce the expression of SLAM in immune cells; then, SLAM amplifies TCR signaling [26, 27]. After tyrosine phosphorylation of the immunoreceptor tyrosine-based switch motif (ITSM) in the intracellular domain of SLAM, the SLAM-SAP-Fyn compound is formed and then initiates intracellular signal transduction [28, 29]. The SLAM-SAP signaling pathway has been reported to induce the synthesis of Th2 cytokines and decrease the Th1 immune response [27]. Moreover, SAP deficiency damages the germinal center and reduces the number of antigen-specific memory B cells and plasma cells [30, 31]. An increase in the expression of SLAM or SAP has been observed in autoimmune diseases and allergic disease [32–35]. In addition, SLAM is a potential target for inflammatory and autoimmune diseases [36].

Because SLAM and SAP have been shown to be important in Tfh-B cell interactions, we asked whether these two molecules play a role in the pathogenesis of GD. We found that in patients with GD, the expression of SLAM on CD19^+^ B cells and SAP in CD4^+^CXCR5^+^ T cells was increased, the proportion of peripheral blood CD4^+^CXCR5^+^Foxp3^+^ Tfr cells was decreased, and costimulation of SLAM along with CD3/TCR stimulation potently decreased the proportion of Tfr cells in vitro. Collectively, our results indicate that the SLAM/SAP signaling pathway may be involved in the pathogenesis of Graves' disease.

## 2. Materials and Methods

### 2.1. Individuals and Samples

All GD patients included in our studies were recruited from the Affiliated People's Hospital of Jiangsu University and newly diagnosed according to clinically and biochemically verified hyperthyroidism. Thirty-five age- and sex-matched healthy individuals were included as controls, including 27 females and 8 males, ranging from 28 to 50 years old. They had no history of GD or any other related diseases. The main clinical data and laboratory test indicators of the patients are shown in Table 1. All indicators were measured by chemiluminescent immunoassays according to the manufacturer's protocol. All blood samples were obtained in accordance with the regulations and approval of the Affiliated People's Hospital of Jiangsu University.

### 2.2. Cell Isolation and Purification

Peripheral blood mononuclear cells (PBMCs) were isolated by density gradient centrifugation over Ficoll-Hypaque solution. Untouched CD4^+^ T cells were purified from PBMCs using a CD4^+^ T Cell Isolation Kit (Miltenyi Biotec GmbH, Bergisch Gladbach, Germany). CD4^+^CXCR5^+^ T cells were purified from CD4^+^ T cells by FITC-conjugated anti-human CXCR5 mAb and anti-FITC MicroBeads (Miltenyi Biotec GmbH, Bergisch Gladbach, Germany). All operations are based on the manufacturer's instructions.

### 2.3. Cell Culture and Stimulation

Ninety-six-well cell culture plates were precoated with 0.5 mg/ml anti-CD3 mAb (clone OKT3; eBioscience, San Diego, CA) for 2 h, and then, purified CD4^+^CXCR5^+^ T cells from healthy control subjects were cultured for 48 h in the plates (1 × 10^5^ cells/well) with 100 *μ*l of culture medium (Miltenyi Biotec) in the presence of anti-SLAM mAb (clone A12 (7D4); BioLegend, San Diego, CA).

### 2.4. RNA Isolation and Real-Time PCR

TRIzol reagent (Invitrogen, Carlsbad, CA) was added to the purified CD4^+^ T cells following the manufacturer's instructions to extract RNA. Then, reverse transcription was performed according to the manufacturer's instructions (Toyobo, Osaka, Japan). Next, real-time PCR was performed in duplicate with Bio-Rad SYBR Green Supermix (Bio-Rad, Hercules, CA). Primer sequences were as follows: SLAM: forward 5′-ACCGTGAGCAACCCTATCAG-3′, reverse 5′-CCCTAACAGCCCAGCATACA-3′; SAP: forward 5′-GGACGCAGTGGCTGTGTAT-3′, reverse 5′-TGGCACGCTCTCGCTGT-3′. Each gene was normalized to *β*-actin with the following primers: forward 5′-CACGAAACTACCTTCAACTCC-3′, reverse 5′-CATACTCCTGCTTGCTGATC-3′. Data were analyzed by Bio-Rad CFX Manager software.

### 2.5. Flow Cytometry Analysis

Anticoagulant blood was stained with FITC-conjugated anti-CD19 (clone HIB19; BioLegend) and phycoerythrin- (PE-) conjugated anti-SLAM (clone A12 (7D4); eBioscience) mAb after lysing red blood cells (BD Pharmingen) to detect the expression of SLAM on CD19^+^ B cells in peripheral blood.

PBMCs were washed and stained with phycoerythrin-cy5-conjugated anti-CD3 (clone OKT3; eBioscience), APC-conjugated anti-CD4 (clone A161A1; BioLegend), and Alexa Fluor 488-conjugated anti-CXCR5 (clone RF8B2; BD Pharmingen) mAb. Half of the cells were stained with PE-conjugated anti-SLAM, and the other half of the cells were stained with PE-conjugated anti-SAP (clone XLP-1D12; eBioscience) after permeabilization (Invitrogen). Other PBMCs were stained with APC-conjugated anti-CD4, Alexa Fluor 488-conjugated anti-CXCR5, and PE-conjugated anti-Foxp3 (clone REA1253; Miltenyi Biotec) mAb to detect the proportion of Tfr cells in peripheral blood.

Cultured CD4^+^CXCR5^+^ T cells in some of the wells were collected and stained with PE-conjugated anti-Foxp3 mAb to detect the proportion of Tfr cells. For detection of cytokines [37], PMA (50 ng/ml) and ionomycin (1 *μ*g/ml) were added to the CD4^+^CXCR5^+^ T cells and cultured for 1 h, and then, BFA (1 *μ*g/ml, for detecting IFN-*γ* and IL-4) and monensin (2 *μ*g/ml, for detecting IL-17) were added and cultured for 5 h. Then, the cells were collected and added with 1 ml IC Fixation Buffer (Invitrogen). After mixing, they were treated at 4°C for 10 min and washed twice with 1 ml 1x Permeabilization (Invitrogen) and then centrifuged at 4°C and 500 g for 5 min each time. The supernatant was discarded and 40 *μ*l 1x Permeabilization was applied to the resuspended cells. Then, the cells were stained with PE-conjugated anti-IFN-*γ* (clone REA600, Miltenyi Biotec), PE-conjugated anti-IL-4 (clone REA895, Miltenyi Biotec), or PE-conjugated anti-IL-17 mAb (clone N49-653, Miltenyi Biotec).

All staining was performed according to the manufacturers' protocol. Isotype-matched mAb controls were used in all procedures. Cells were analyzed with a FACSCalibur flow cytometer (Becton Dickinson, Sparks, MD) and BD Biosciences Accuri C6, and the results were analyzed with WinMDI 2.9 software or Accuri C6.

### 2.6. Statistical Analysis

Two-tailed Student's *t*-test was applied for statistical comparison of two groups. Correlations between variables were determined by Spearman rank test. Data are expressed as the mean ± SD. A *P* value of 0.05 or less was considered significant. Data were analyzed with GraphPad Prism 6 software.

## 3. Results

### 3.1. The Expression of SLAM and SAP in the Peripheral Blood of Patients with GD

**Figure 1:**
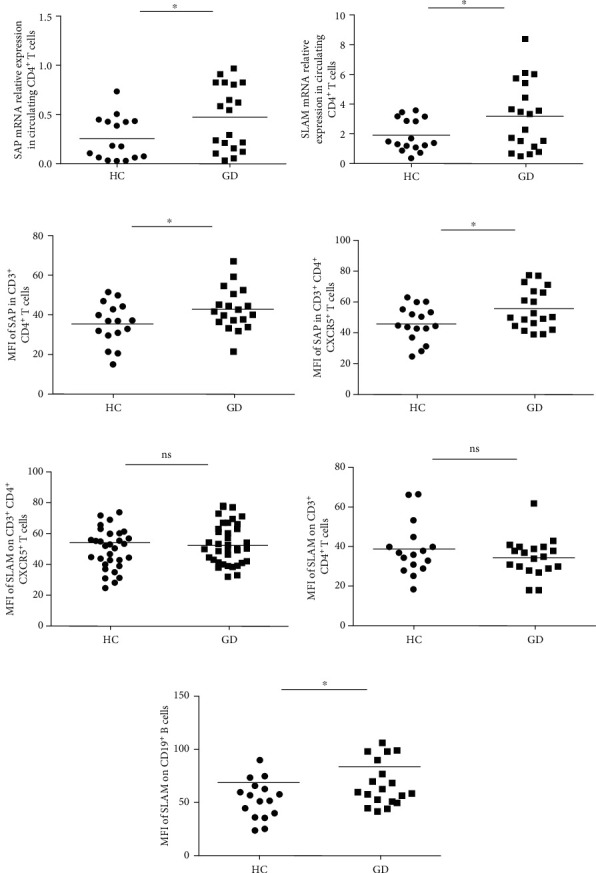
The expression of SLAM and SAP in the peripheral blood of the patients with GD and the healthy controls. The levels of (a) SAP mRNA and (b) SLAM mRNA in circulating CD4^+^ T cells from the patients with GD and the healthy controls. The mean fluorescence intensity (MFI) of SAP was analyzed by flow cytometry (FCM) in circulating (c) CD4^+^ T cells and (d) CD4^+^CXCR5^+^ T cells from the patients with GD and the healthy controls. The MFI of SLAM was analyzed by FCM on circulating (e) CD4^+^ T cells and (f) CD4^+^CXCR5^+^ T cells from the patients with GD and the healthy controls. (g) The MFI of SLAM was analyzed by FCM on circulating CD19^+^ B cells from the patients with GD and the healthy controls. Each data point represents an individual subject; horizontal lines show the mean. ^∗^*P* < 0.05, *n* = 19; ns: no significant differences.

Many studies have reported that SLAM or SAP is elevated in some autoimmune diseases; thus, we investigated whether the expression of these two molecules is changed in patients with GD. We first detected the levels of SAP mRNA and SLAM mRNA in circulating CD4^+^ T cells and found that both the SAP mRNA level and the SLAM mRNA level were increased significantly in the patients with GD compared to the healthy controls (Figures 1(a) and 1(b)). Then, we analyzed the mean fluorescence intensity (MFI) of SAP in CD3^+^CD4^+^ T cells and CD3^+^CD4^+^CXCR5^+^ T cells from PBMCs and found that the expression of SAP was substantially enhanced in these two subsets of cells from the patients with GD compared with the healthy controls (Figures 1(c) and 1(d)). Subsequently, we compared the MFI of SLAM on the surface of CD3^+^CD4^+^ T cells and CD3^+^CD4^+^CXCR5^+^ T cells, but there was no significant difference between the patients with GD and the healthy controls (Figures 1(e) and 1(f)).

Studies have shown that the interaction between SLAM-SLAM on T cells and B cells plays an important role in humoral immunity, so we analyzed the MFI of SLAM on circulating CD19^+^ B cells. As shown in Figure 1(g), the level of SLAM on CD19^+^ B cells in the patients with GD was higher than that in the healthy controls.

### 3.2. The Role of SLAM on Circulating CD4^+^CXCR5^+^ T Cells In Vitro

**Figure 2:**
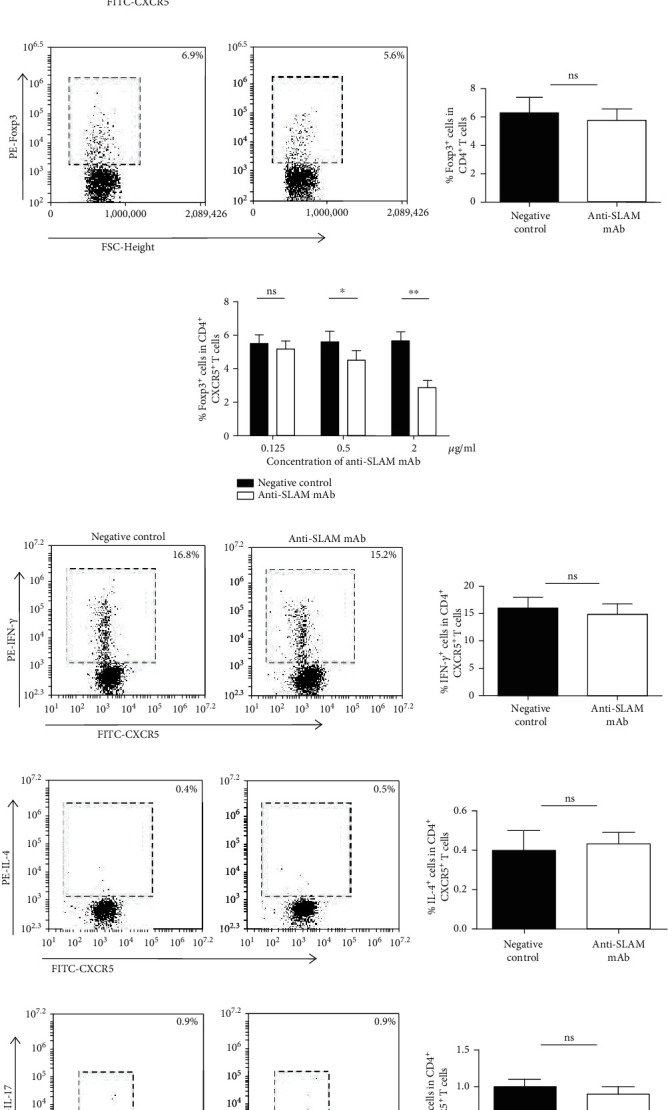
The role of SLAM on circulating CD4^+^CXCR5^+^ T cells. Addition of 0.5 *μ*g/ml anti-CD3 mAb and 2 *μ*g/ml anti-SLAM mAb to CD4^+^CXCR5^+^ T cells, followed by determination of the percentage of (a) Foxp3^+^ cells, (d) INF-*γ*^+^ cells, (e) IL-4^+^ cells, and (f) IL-17^+^ cells. (b) Addition of 0.5 *μ*g/ml anti-CD3 mAb and 2 *μ*g/ml anti-SLAM mAb to CD4^+^ T cells and detection of the percentage of Foxp3^+^ cells. (c) Addition of 0.5 *μ*g/ml anti-CD3 mAb with 0.125 *μ*g/ml, 0.5 *μ*g/ml, and 2 *μ*g/ml anti-SLAM mAb to CD4^+^CXCR5^+^ T cells and detection of the percentage of Foxp3^+^ cells. Each experiment was repeated three times; ^∗∗^*P* < 0.01; ^∗^*P* < 0.05; ns: no significant differences.

Because the expression of SLAM on CD19^+^ B cells and SAP in CD4^+^CXCR5^+^ T cells was both highly expressed in GD, we wondered whether these increased SLAM molecules on CD19^+^ B cells affect circulating CD4^+^CXCR5^+^ T cells. Therefore, we used anti-SLAM mAb with anti-CD3 mAb to stimulate CD4^+^CXCR5^+^ T cells in vitro. After stimulation, flow cytometry was used to analyze the proportion of IFN-*γ*^+^ cells, IL-4^+^ cells, IL-17^+^ cells, and Foxp3^+^ cells in the CD4^+^CXCR5^+^ T cells. The proportion of Foxp3^+^ cells in CD4^+^CXCR5^+^ T cells declined sharply after stimulation, and this reduction caused by anti-SLAM mAb occurred in a concentration-dependent manner (Figures 2(a) and 2(c)). However, the percentage of Foxp3^+^ cells in the CD4^+^ T cells remained unaltered after treatment with anti-SLAM mAb together with anti-CD3 mAb (Figure 2(b)). In addition, the proportion of IFN-*γ*^+^ cells, IL-4^+^ cells, and IL-17^+^ cells showed almost no change after treatment with anti-SLAM mAb (Figures 2(d)–2(f)).

### 3.3. The Percentage of Tfr Cells Decreased in the Peripheral Blood from the Patients with GD

**Figure 3:**
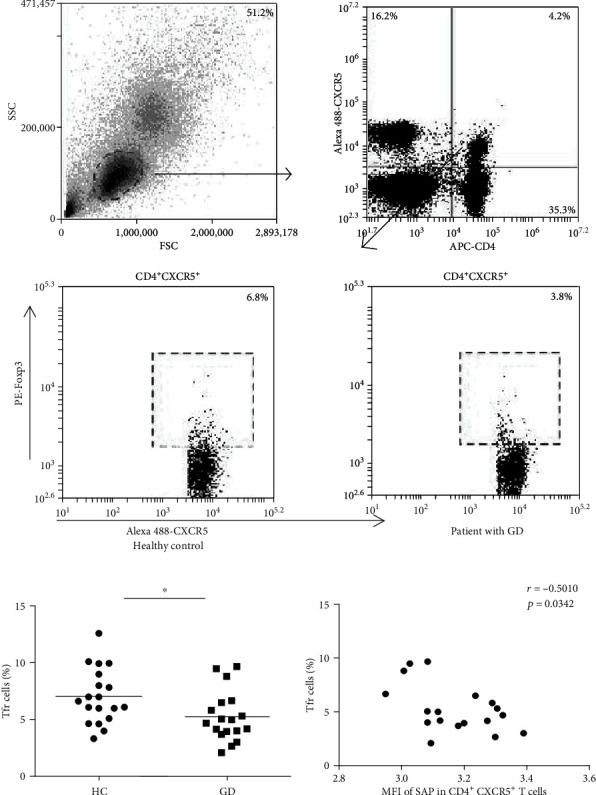
The percentage of Tfr cells in patients with GD. (a) Representative dot plots of CD4^+^CXCR5^+^Foxp3^+^ Tfr cells. (b) The percentage of Tfr cells from the patients with GD was lower than that in the healthy controls. (c) A negative correlation was found between the percentage of Tfr cells and the expression of SAP in CD4^+^CXCR5^+^ T cells from the patients with GD. ^∗^*P* < 0.05, *n* = 18.

As anti-SLAM mAb could reduce the ratio of Tfr cells in vitro, we next analyzed the proportion of Tfr cells in the patients with GD. We gated CD3^+^CD4^+^CXCR5^+^ T cells in PBMCs and then identified Foxp3^+^ T cells as Tfr cells (Figure 3(a)). As shown in Figure 3(b), the proportion of Tfr cells decreased in the patients with GD compared with the healthy controls. Further, the proportion of Tfr cells was negatively correlated with SAP expression in the patients with GD (Figure 3(c)).

### 3.4. The Correlations between Tfr Cells and Autoantibodies

**Figure 4:**
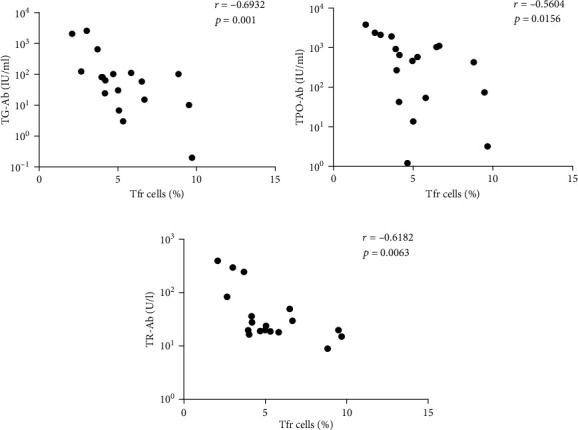
The correlations between the proportion of Tfr cells in PBMCs and the levels of (a) TR-Ab, (b) TPO-Ab, and (c) TG-Ab in serum from the patients with GD.

TR-Ab, TG-Ab, and TPO-Ab are critical autoantibodies that provide evidence for diagnosis of GD, so we intended to analyze the relationship between the proportion of Tfr cells in peripheral blood and the levels of autoantibodies. The results showed that there were negative correlations between the proportion of Tfr cells in PBMCs and the serum concentrations of TR-Ab, TPO-Ab, and TG-Ab in the patients with GD (Figure 4).

## 4. Discussion

Graves' disease is a kind of autoimmune disease in which humoral immunity predominates. Patients produce a variety of autoantibodies to stimulate the proliferation of thyroid follicular cells and then increase the secretion of thyroid hormones, eventually leading to multiple system syndrome. Therefore, autoantibodies in patients with GD are key factors in the pathogenesis of this disease, but the specific pathogenesis has not been fully elucidated. In recent years, researchers have found that the existence of Tfh cells is advantageous to the formation and maintenance of germinal centers and helps B cells differentiate into plasma cells and memory B cells. Tfh-B cell-mediated humoral immunity plays an important role in the pathogenesis of GD. The SLAM-SAP signaling pathway is one of the most important signaling pathways in Tfh cells and plays an important role in Tfh-B cell interactions [38, 39]. It has been reported that SLAM or SAP is increased in autoimmune diseases [32–34], and thus, we investigated whether SLAM and SAP would be increased in GD. First, we analyzed the expression of SLAM and SAP on CD4^+^ T cells and CD4^+^CXCR5^+^ T cells in peripheral blood and found that the level of SAP in the CD4^+^ T cells and the CD4^+^CXCR5^+^ T cells was increased in the patients with GD. Although the SLAM mRNA level was significantly increased in CD4^+^ T cells from GD patients, but there was no significant difference in SLAM protein on CD4^+^ T cells or CD4^+^CXCR5^+^ T cells in peripheral blood between the patients with GD and the healthy controls. Since SLAM on T cells and B cells acts as a self-ligand, we then detected the level of SLAM on CD19^+^ B cells. Our results showed that the level of SLAM on CD19^+^ B cells was increased in the patients with GD.

Next, we explored whether the elevated SLAM on CD19^+^ B cells had any influence on CD4^+^CXCR5^+^ T cells, which express high levels of SAP. As early as 1995, researchers used anti-SLAM mAb to stimulate human PBMCs and found that anti-SLAM mAb could activate T cells and promote T cell proliferation [40]. Researchers found that mice with SLAMF1/5/6 deficiency showed enhanced humoral immune responses. Although this study suggests that the SLAM molecule may negatively regulate the humoral immune response, there is no specific result showing that a single defect of the SLAMF1 molecule plays a role in the humoral immune response [41]. In addition, we were interested in the studies on SLAMF1 regulating Treg cells, and one of these studies found that the proliferation of Treg cells induced by alloantigen could be inhibited by anti-SLAMF1 mAb [42]. Does this signaling pathway affect Tfr cells? To address this question, we stimulated CD4^+^CXCR5^+^ T cells with anti-SLAM mAb in vitro. As expected, the anti-SLAM mAb decreased the proportion of Tfr cells in a concentration-dependent manner, but it did not affect the proportion of Tfh1 cells, Tfh2 cells, or Tfh17 cells. Furthermore, although anti-SLAM mAb could decrease the proportion of CD4^+^CXCR5^+^Foxp3^+^ Tfr cells, it did not affect the proportion of CD4^+^Foxp3^+^ Treg cells.

Tfr cells are developed from Treg cells, which are present in the germinal center, and play a role in inhibiting the reaction of the germinal center, limiting the number of Tfh cells and reducing the production of antibodies. Many studies have shown that Tfh/Tfr disorders play an important role in the pathogenesis of autoimmune diseases, and the correction of Tfh/Tfr may become a new way to treat autoimmune diseases [16, 43]. It has been reported that in the BXD2 mouse model of autoimmune disease, IL-21 increased the percentage of Tfh/Tfr cells and the number of Tfh cells to promote the formation of the germinal center; accordingly, the number of Tfr cells increased in IL-21-deficient BXD2 mice, and Tfr cells transferred to BXD2 mice can reduce the formation of the germinal center and autoantibody production [16]. Although Tfr cells are considered a kind of cell that suppresses the immune response, Tsuji et al. found that Foxp3^+^ T cells could be transformed into Tfh cells by the loss of Foxp3 and then induce the formation of a germinal center and the generation of IgA in Peyer's patches of the mouse intestine [43]. Another study reported that the percentage of Tfr cells in peripheral blood from child patients with idiopathic thrombocytopenic purpura (ITP) was decreased; in contrast, Tfh cells were increased, and the proportion of Tfr/Tfh cells was reduced, which was associated with disease progression [44]. These results suggest that Tfh cells and Tfr cells have a complex and intimate connection. Since we found that adding anti-SLAM mAb to CD4^+^CXCR5^+^ T cells might decrease the proportion of Tfr cells and the previous results indicated that peripheral blood Tfh cells increased in GD patients, what is the effect on Tfr cells? Ultimately, our results showed that the proportion of peripheral blood Tfr cells considerably declined in the patients with GD. In addition, the proportion of Tfr cells was negatively correlated with the SAP level in CD4^+^CXCR5^+^ T cells. All of the above results indicate that increased SLAM/SAP may be one of the reasons for the Tfr cell reduction in patients with GD.

Our results showed that the levels of SLAM on CD19^+^ B cells and SAP in CD4^+^CXCR5^+^ T cells were increased, SLAM may decrease the proportion of Tfr cells, and the proportion of Tfr cells correlates with disease severity. These data extend the understanding of the pathogenesis of GD and identify a potential biomarker. Further studies should focus on the mechanism of SLAM/SAP signaling pathways in Tfr cells.

## Figures and Tables

**Table 1:** Clinical features of the patients with GD and healthy controls included in this study.

	Patients with GD	Healthy controls	Range
Gender (M/F)	5/32	8/27	
Age (year)	40.81 ± 13.13	37.13 ± 11.89	
FT3 (pmol/l)	11.22 ± 9.00	4.15 ± 1.23	3.10-6.00
FT4 (pmol/l)	25.13 ± 13.19	12.78 ± 4.11	7.86-17.41
TSH (uIU/ml)	0.03 ± 0.02	3.34 ± 1.18	0.34-5.60
TR-Ab (U/l)	75.39 ± 115.04	13.58 ± 11.70	0-30
TPO-Ab (IU/ml)	917.91 ± 1099.29	3.70 ± 3.19	0-9
TG-Ab (IU/ml)	329.83 ± 718.06	2.39 ± 1.34	0-4

Data correspond to the arithmetic mean ± SD. M: male; F: female.

## Data Availability

The data supporting the conclusions of this article are included in the article.
